# Bioinformatics and Microarray Analysis of miRNAs in Aged Female Mice Model Implied New Molecular Mechanisms for Impaired Fracture Healing

**DOI:** 10.3390/ijms17081260

**Published:** 2016-08-03

**Authors:** Bing He, Zong-Kang Zhang, Jin Liu, Yi-Xin He, Tao Tang, Jie Li, Bao-Sheng Guo, Ai-Ping Lu, Bao-Ting Zhang, Ge Zhang

**Affiliations:** 1 Institute for Advancing Translational Medicine in Bone & Joint Diseases, School of Chinese Medicine, Hong Kong Baptist University, Hong Kong, China; hebinghb@gmail.com (B.H.); liujin_hkbu@163.com (JinL.); berry.he@gmail.com (Y.-X.H.); boris.g.guo@gmail.com (B.-S.G.); 2 Institute of Integrated Bioinformedicine & Translational Science, HKBU Shenzhen Research Institute and Continuing Education, Shenzhen 518000, China; 3 School of Chinese Medicine, Faculty of Medicine, The Chinese University of Hong Kong, Hong Kong, China; maxzhangzk@cuhk.edu.hk (Z.-K.Z.); lijie_bio@126.com (JieL.); 4 Department of Obstetrics and Gynaecology, Faculty of Medicine, The Chinese University of Hong Kong, Hong Kong, China; tangtao@cuhk.edu.hk

**Keywords:** impaired fracture healing, bioinformatics, miRNA

## Abstract

Impaired fracture healing in aged females is still a challenge in clinics. MicroRNAs (miRNAs) play important roles in fracture healing. This study aims to identify the miRNAs that potentially contribute to the impaired fracture healing in aged females. Transverse femoral shaft fractures were created in adult and aged female mice. At post-fracture 0-, 2- and 4-week, the fracture sites were scanned by micro computed tomography to confirm that the fracture healing was impaired in aged female mice and the fracture calluses were collected for miRNA microarray analysis. A total of 53 significantly differentially expressed miRNAs and 5438 miRNA-target gene interactions involved in bone fracture healing were identified. A novel scoring system was designed to analyze the miRNA contribution to impaired fracture healing (RCIFH). Using this method, 11 novel miRNAs were identified to impair fracture healing at 2- or 4-week post-fracture. Thereafter, function analysis of target genes was performed for miRNAs with high RCIFH values. The results showed that high RCIFH miRNAs in aged female mice might impair fracture healing not only by down-regulating angiogenesis-, chondrogenesis-, and osteogenesis-related pathways, but also by up-regulating osteoclastogenesis-related pathway, which implied the essential roles of these high RCIFH miRNAs in impaired fracture healing in aged females, and might promote the discovery of novel therapeutic strategies.

## 1. Introduction

Fracture healing is a complex process, which is impacted profoundly by aging and osteoporosis [1,2]. Approximately one half of women aged 60 years or older are found to have osteoporosis [3], and over 50% of postmenopausal women will suffer an osteoporotic fracture [4]. Due to the impaired capacity of fracture repair, the incident of fracture nonunion or delayed union is high in aged women [5], leading to increased morbidity and mortality as well as high cost of caring for patients [4,6,7,8]. To date, the underlying mechanism responsible for the impaired fracture healing in aged women remains underexplored. Hence, it is highly desirable to understand the differences in the molecular and cellular events during the fracture healing progress between adult and aged women.

MicroRNAs (miRNAs) are small non-coding RNAs of ~22 nucleotides which function as key post-transcriptional gene expression regulators by targeting the 3′-UTR of the mRNAs [9]. The miRNAs are important regulators of many key biological processes, such as cell proliferation, differentiation, and organ development [9]. They are also associated with human disease, including cancer [10]. In the field of skeletal biology, growing evidence suggests that miRNAs are key regulators of bone modeling and remodeling including angiogenesis [11,12], chondrogenesis [13,14], osteogenesis [14], and osteoclastogenesis [15,16,17], and they also participate in the regulation of fracture healing [11,18]. The fracture healing process is typically constructed by four stages, including initial inflammatory reaction, formation of soft callus formation, formation of hard callus, and remodeling to original bone contour [19]. Angiogenesis, chondrogenesis, osteogenesis, and osteoclastogenesis are essential components of fracture healing, while the impairment of these components has been reported to induce improper fracture healing [20,21,22,23]. Therefore, studying miRNAs during fracture healing will help identifying novel therapeutic targets for improving impaired fracture healing in aged women.

Currently, fold change and *t*-test are common methods used to identify differentially expressed miRNAs in bone fracture [24,25]. Usually, dozens to hundreds of differentially expressed miRNAs are identified in these studies. It is hard to judge which differentially expressed miRNA is more important than others for fracture healing. Therefore, an algorithm scoring the contribution of every differentially expressed miRNA to fracture healing is needed. 

In this study, the miRNA expression profiles in fracture healing of adult and aged female mice were examined by microarray analysis. Differentially expressed miRNAs were identified between adult and aged female mice, followed by identification of target genes using an integrated method. We designed a novel bioinformatics scoring system to analyze the contribution of differentially expressed miRNAs to impaired fracture healing (RCIFH). Furthermore, pathway enrichment analyses were performed for the differentially expressed miRNAs with high RCIFH values to analyze their potential roles in impaired fracture healing of aged female mice.

## 2. Results

### 2.1. Fracture Healing Is Impaired in Aged Female Mice

**Figure 1:**
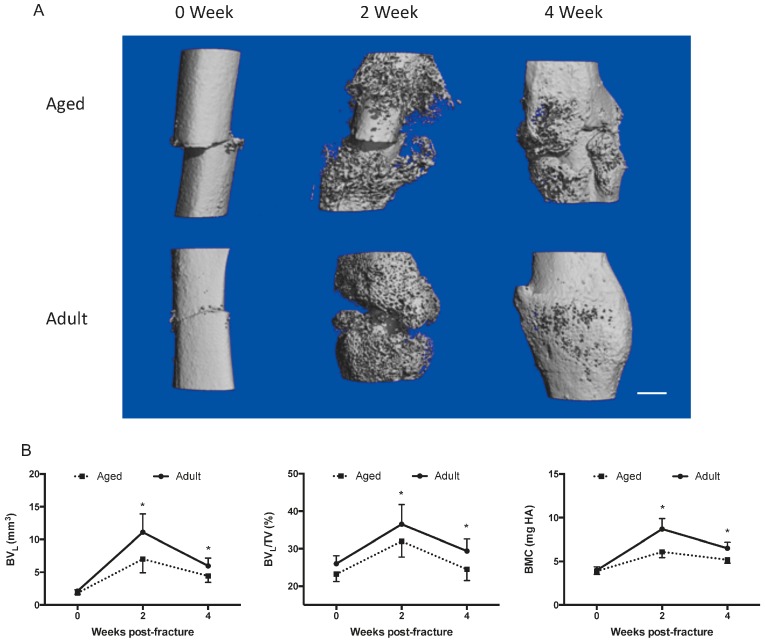
Fracture healing in aged and adult female mice (**A**) representative three-dimensional (3D) images of the fracture calluses in adult and aged groups at each time point after fracture; (**B**) time course changes in low-density bone volume (BV_L_) (**left**), low-density bone volume fraction (BV_L_/TV) (**middle**), and bone mineral content (BMC) (**right**) of the callus after fracture in each group; *n* = 3. Note: scale bar = 1 mm. * *p* < 0.05 for “Aged” vs. “Adult”.

The micro computed tomography (micro-CT) images demonstrated remarkably different morphologies of the fracture callus between the aged and adult groups. The aged mice showed a smaller amount of newly mineralized callus at 2-week post-fracture and delayed bridging of the fracture site at 4-week post-fracture when compared to adult mice (Figure 1A). Quantitatively, although the bone volume (BV_L_), low-density bone volume fraction (BV_L_/TV), and bone mineral content (BMC) in both groups increased from 0- to 2-week after fracture and decreased gradually thereafter, the above three micro-CT parameters in aged female mice were all significantly lower than those in adult ones at 2- and 4-week post-fracture, respectively (Figure 1B). These evidences indicated that fracture healing was impaired in aged female mice.

### 2.2. MicroRNA (miRNA) Expression Profiles

**Figure 2:**
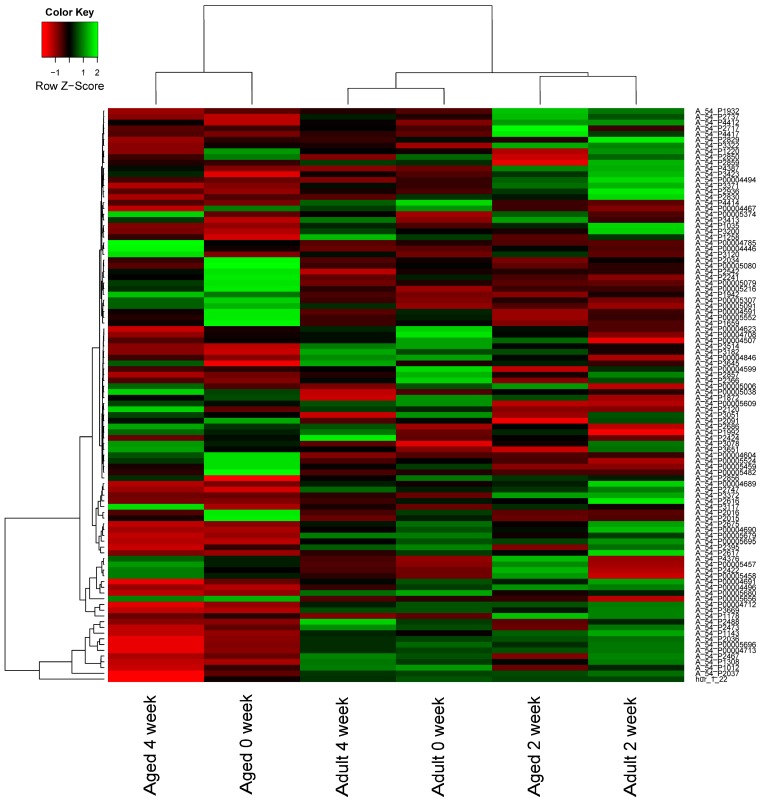
Heat map of differentially expressed microRNAs (miRNAs) in fracture healing. Red represents up-regulation and green represents down-regulation.

The expression of total 1079 mouse miRNAs was analyzed on a mouse SurePrint G3 miRNA Microarray. A total of 53 significantly differentially expressed miRNAs in bone fracture healing were identified. At 2-week post-fracture, 35 miRNAs were identified that were differentially expressed in aged female mice (17 up-regulated and 18 down-regulated), while there were 33 differentially expressed miRNAs in the adult group (23 up-regulated and 10 down-regulated). At 4-week post-fracture, 10 miRNAs were identified that were differentially expressed in aged female mice (5 up-regulated and 5 down-regulated) while there were 6 differentially expressed miRNAs in the adult group (4 up-regulated and 2 down-regulated) (Supplementary Data S1). As shown in Figure 2, the miRNA expression patterns were different between adult and aged mice during fracture healing.

### 2.3. Target Genes and Molecular Network Construction

**Figure 3:**
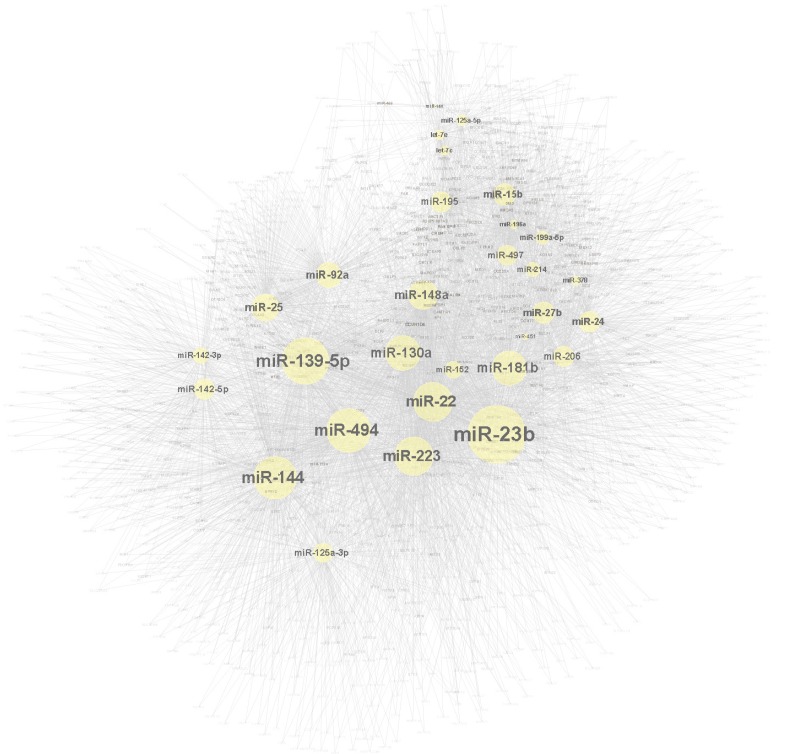
miRNA-target gene interactions for the significantly differentially expressed miRNAs in bone fracture healing.

A total of 210,365 miRNA-target interactions were predicted for differentially expressed miRNAs (|log_2_FC| > 0). They were included in further selection using gene expression data of angiogenesis, chondrogenesis, and osteogenesis. Since miRNAs target genes and inhibit their expression, the expression pattern of a miRNA and its target gene should be opposite. Therefore, for up-regulated miRNAs in bone fracture healing, down-regulated target genes were selected, while for down-regulated miRNAs, up-regulated target genes were selected. Finally, 43,839 miRNA-target interactions were predicted to be involved in bone fracture healing, in which 5438 miRNA-target interactions are for the 53 significant differentially expressed miRNAs (Figure 3).

### 2.4. miRNA Contribution to Impaired Fracture Healing (RCIFH) and Pathway Enrichment Analysis

**Figure 4:**
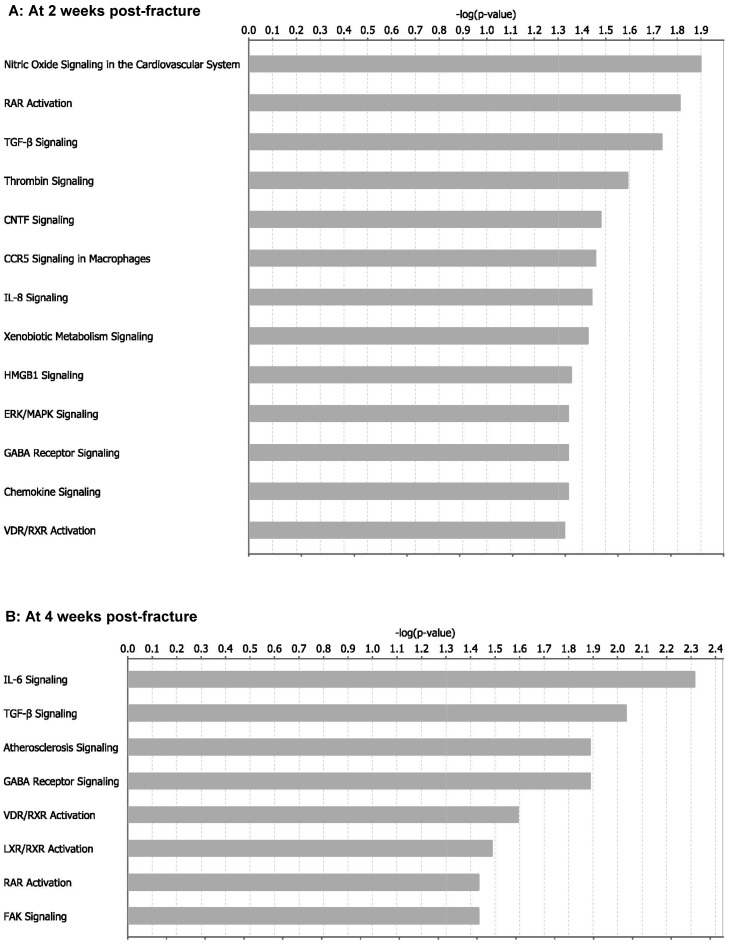
Significantly enriched pathways of top 100 highest miRNA contribution to impaired fracture healing (RCIFH) miRNA targets at (**A**) 2-week after fracture and (**B**) 4-week after fracture.

**Figure 5:**
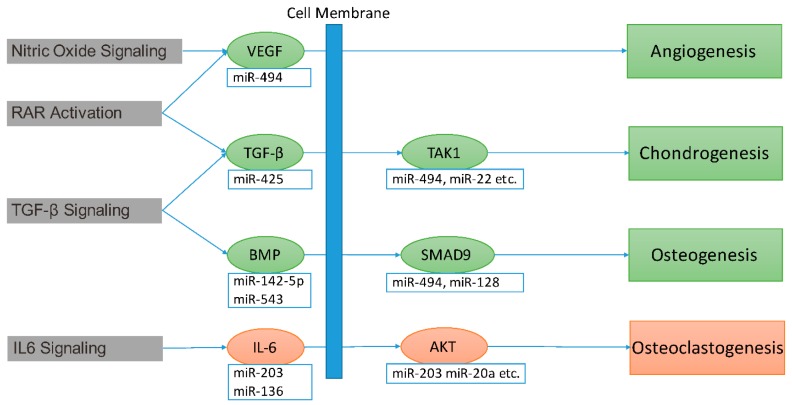
The effect of top 100 highest RCIFH miRNAs on the key genes of nitric oxide signaling, retinoid acid receptor (RAR) activation, transforming growth factor-β (TGF-β) signaling, and interleukin 6 (IL-6) signaling pathways in impaired fracture healing. Red: gene expression and function are activated in aged female mice compared to adult ones. Green: gene expression and function are inhibited in aged female mice compared to adult ones.

The contribution of every differentially expressed miRNA to impaired fracture healing (RCIFH) was calculated in the context of molecular network and biological progresses using the algorithms described in the Materials and Methods section. The absolute value of RCIFH reveals the impact of the differentially expressed miRNA on fracture healing. A positive RCIFH indicates that the miRNA improves bone fracture healing, while a negative RCIFH indicates the miRNA impairs bone fracture. The top 10 RCIFH miRNAs at 2- or 4-week are presented in Table 1, more detail information is listed in Supplementary Data S1. Among these miRNAs, miR-142-5p [26], miR-223 [27], miR-22 [28,29] miR-24 [17], miR-497 [30], and miR-195 [30] have been found to participate fracture healing. The other 11 miRNAs are novel findings by this study. According to the RCIFH analysis, miR-494 shows to be the most important miRNA to impair fracture bone fracture healing at both 2- and 4-week post-fracture (Table 1). To investigate how much RCIFH miRNAs impact fracture healing, target genes with the 100 highest RCIFH miRNAs were analyzed (Supplementary Data S2). At 2-week after fracture, target genes of high RCIFH miRNAs were significantly enriched (*p*-value < 0.05) in 13 pathways (Figure 4A), while at 4-week after fracture, target genes were significantly enriched (*p*-value < 0.05) in 8 pathways (Figure 4B). These pathways are mainly involved in angiogenesis-, chondrogenesis-, osteogenesis-, and osteoclastogenesis-related functions, which are important for fracture healing. Interestingly, the top RCIFH miR-494 may impair fracture healing in aged females by inhibiting angiogenesis, chondrogenesis, and osteogenesis (Figure 5).

### 2.5. The miR-494 Inhibits Chondrogenic Differentiation in Vitro

**Figure 6:**
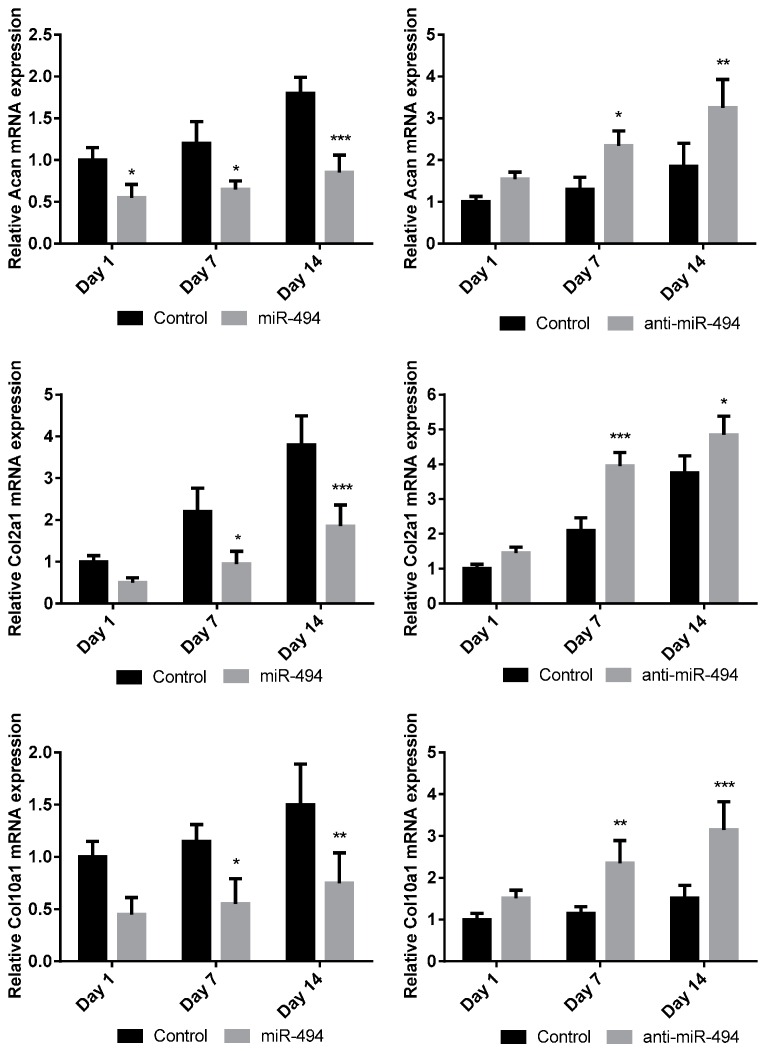
The miR-494 inhibits chondrogenic differentiation in C3H10T1/2 cells. The miR-494, anti-miR-494, or inactive controls were transfected into C3H10T1/2 cells. Expressions of chondrogenic differentiation markers (Acan, Col2a1, and Col10a1) were detected by QPCR at day 1, 7, and 14 after transfection. Note: Data was represented as mean ± SD, * *p* < 0.05, ** *p* < 0.01, *** *p* < 0.001.

As mentioned above, the miR-494 is the miRNA with the top RCIFH and was predicted to impair fracture healing by inhibiting angiogenesis, chondrogenesis, and osteogenesis. Previous studies have shown that miR-494 inhibits angiogenesis [31,32]. To further investigate whether miR-494 has an impact on the progress of chondrogenic differentiation, miR-494 mimics or anti-miR-494 were transfected into C3H10T1/2 cells. The chondrogenic differentiation of C3H10T1/2 cells was induced by medium containing transforming growth factor-β (TGF-β3). Thereafter, according to the quantitative real-time polymerase chain reaction (QPCR) analysis, chondrogenesis markers—including Acan, Col2a1 and Col10a1—were significantly decreased at the mRNA level in C3H10T1/2 cells transfected with miR-494 mimics (Figure 6). Consistently, anti-miR-494, using the same induction conditions as mentioned above, enhanced the chondrogenic differentiation of C3H10T1/2 cells, as evidenced by significant increases of chondrogenesis markers at the mRNA level (Figure 6). These results indicated that miR-494 inhibits chondrogenic differentiation in vitro.

## 3. Discussion

In order to extend our understanding on the pathogenesis of impaired fracture healing in aged females, this study focused on comparing the miRNA expression profiles at fracture site between adult and aged female mice during fracture healing. The differentially expressed miRNAs were identified and their dynamic expression patterns were described. A novel bioinformatics scoring system was designed to analyze the contribution of every differentially expressed miRNA to impaired fracture healing (RCIFH).

In this study, fracture healing was remarkably impaired in aged female mice, as evidenced by the micro-CT data showing a smaller amount of newly mineralized calluses at the early stage and delayed bridging of the fracture gap at the later stage. Additionally, lower micro-CT parameters were at the fracture site during bone healing in the aged female mice when compared to the adult ones. Bone healing after fracture is a complicated and sequential process including inflammation, angiogenesis, progenitor cell recruitment, chondrogenesis, osteogenesis, and osteoclastogenesis [19,20,33,34]. In the aged population, the processes of angiogenesis, chondrogenesis, and osteogenesis have been down-regulated [35,36], whereas the process of osteoclastogenesis has been up-regulated [37], which results in impaired fracture healing. However, the regulatory mechanisms underlying these changes still have not been fully understood.

The miRNAs are important regulatory molecules, which have been demonstrated to regulate angiogenesis, chondrogenesis, osteogenesis, and osteoclastogenesis by inhibiting gene expression [38,39,40]. The importance of miRNA on fracture healing highly relies on its impact on target genes, which cannot be fully illustrated by its own expression change. Sometimes, the most differentially expressed miRNA might not have the strongest impact on impaired fracture healing in the aged female mice. Therefore, we designed the RCIFH method to calculate the impact of miRNAs on impaired fracture healing. RCIFH equals fold changes difference between adult and aged group times the network power of the miRNA, which has more significance than differential expression only. A miRNA with a positive RCIFH means this miRNA improves bone fracture healing, while a negative RCIFH reveals the miRNA impairs bone fracture. Usually, the RCIFH value indicates the importance of the miRNA to the impaired fracture healing. According to the RCIFH in present study, miR-494 has been the most important miRNA at both 2- and 4-week post-fracture (Table 1). MiR-494 has been found to inhibit the vascular endothelial growth factor (VEGF), an angiogenic factor, in vitro [31], and the inhibition of miR-494 could increase neovascularization [32]. An angiogenesis-related signaling pathway—nitric oxide signaling—is the most significantly suppressed pathway at 2-week after fracture, indicating the suppression of angiogenesis in aged female mice might be one of the major reasons for impaired fracture healing, and miR-494 might play some role in this process (Figure 5). Our study further revealed that the miR-494 inhibits chondrogenic differentiation in vitro. The pathway analysis indicated that miR-494 inhibits genes in the retinoid acid receptor (RAR) pathway, which plays a fundamental role in chondrogenesis [41,42]. These results indicate the potential role of miR-494 in inhibiting chondrogenesis during fracture healing in aged female mice.

In order to investigate molecular mechanisms underlying high RCIFH miRNAs in impaired fracture healing, we analyzed target genes with the top 100 RCIFH miRNAs. Although strict criteria have been used to increase the confidence of the predicted miRNA-target interactions in fracture healing, false positive miRNA-target interaction may still exist. Since there are more than 15,000 miRNA-target interactions predicted, as listed in Supplementary Data S2, we performed further functional analysis at the pathway level to eliminate the influence of scattered false positive miRNA-target interactions. 

Among these pathways, transforming growth factor-β (TGF-β) signaling and interleukin 6 (IL-6) signaling are most relevant to osteogenesis [43,44]. The target genes of miRNAs were significantly enriched in TGF-β signaling pathway at both 2-week and 4-week post-fracture. TGF-β and bone morphometric protein-2 (BMP-2) are the core signal proteins of the TGF-β signaling pathway [43]. In the present study, TGF-β is predicted to be inhibited by miR-425 at 2-week post-fracture. BMP-2 is predicted to be inhibited by miR-142-5p at 2- and 4-week post-fracture. In addition, the downstream markers of TGF-β signaling—including SMAD family member 9 (SMAD9) and transforming growth factor β-activated kinase 1 (TAK1)—were inhibited by the top RCIFH miRNA, miR-494 (Figure 5). These results indicate that the TGF-β signaling is modulated in aged female mice by the miRNAs. TGF-β signaling pathway could promote osteoblast differentiation and bone formation [43]. Therefore, the inhibition of TGF-β signaling mediated by miRNA might impair fracture healing in aged female mice. On the other hand, the IL-6 signaling pathway was the most significant pathway at 4-week after fracture. IL-6 signaling has been demonstrated to promote osteoclast differentiation [45,46,47], and inhibit osteoblast differentiation [44,48] and bone formation [49,50]. IL-6 negatively regulates osteoblast differentiation through phosphoinositide 3-kinase (PI3K)/Akt pathway [48]. Both genes of IL-6 and its downstream target, Akt, were inhibited by miR-203. Since miR-203 was down-regulated at 4-week after fracture in aged female mice, IL-6 signaling was activated in aged female mice at 4-week after fracture (Figure 5). The miRNA-mediated enhanced bone resorption at the later stage of fracture healing might also be one of the underlying mechanisms of fracture healing impairment in aged female mice.

In summary, we identified differentially expressed miRNAs during impaired fracture healing in aged female mice. Additionally, we designed a novel RCIFH method to analyze the importance of differentially expressed miRNAs to impaired fracture healing. This method not only found miRNAs that are known to participate in bone fracture healing, but also identified novel candidate miRNAs that impair fracture healing. These high RCIFH miRNAs might influence the process of angiogenesis, chondrogenesis, osteogenesis, and, especially, osteoclastogenesis, thereby contributing to the impaired fracture healing in aged female mice. These results indicate clues for a deeper understanding of molecular mechanisms underlying high RCIFH miRNAs involved in impaired fracture healing. Moreover, the novel candidate miRNAs would be potential therapeutic targets for impaired fracture healing in aged women. In the future, further experimental investigations are required to promote the therapeutic strategies for impaired fracture healing.

## 4. Materials and Methods

### 4.1. Animal Model and Micro Computed Tomography (Micro-CT) Analysis

Nine 24-month-old ovariectomized C57BL/6J female mice (Aged Group) and nine 3-month-old C57BL/6J female mice (Adult Group) were recruited in the study. All animal studies were performed in accordance with the guideline from the Animal Experimentation Ethics Committee of the Chinese University of Hong Kong (Hong Kong, China), and were approved by this committee (Reference No. 09/001/GRF). The animals underwent a transverse fracture in right femur. Briefly, prior to fracture induction, intramedullary fixation was used to stabilize the right femur. A small incision lateral to the patella was used to perform the retrograde nailing. The femoral notch was exposed using blunt dissection. Through the proximal metaphyseal zone, a 27 G needle was inserted into the intramedullary canal as described previously [51]. Then cannula was shortened under the cartilaginous surface. Simple interrupted sutures were used to close the wound. A standardized blunt guillotine device was used to induce the transverse femoral fracture. In vivo micro-CT analysis using vivaCT 40 (Scanco Medical, Brüttisellen, Switzerland) was performed at 0-, 2- and 4-week post-fracture, respectively. The contoured regions of interest (ROI) were selected from two-dimensional (2D) CT images. A low-pass Gaussian filter was used to do the three-dimensional (3D) reconstructions of mineralized tissues (Sigma = 1.2, Support = 2). To distinguish the newly mineralized callus from the old cortices, different thresholds (low attenuation = 130, high attenuation = 220) were determined in 2D images using the established evaluation protocol to reconstruct the low- and high-density mineralized tissues [52]. The low-density tissues indicated newly formed callus, while the high-density tissues indicated old cortices and highly mineralized callus. The quantitative analyses were performed covering the middle 400 slices. Morphometric parameters include low-density bone volume (BV_L_, mm^3^), low-density bone volume fraction (BV_L_/TV, %), and bone mineral content (BMC, mgHA) were calculated as indicators of callus mineralization.

### 4.2. Tissue Sample and RNA Isolation

After 0-, 2- and 4-week post-fracture, three mice in each group were sacrificed, respectively. After sacrifice, callus from the right femurs were collected for RNA extraction. Since there was no callus at 0-week post-fracture, the tissue harvested from animals at 0-week was the fracture haematoma present at the fracture site at 3-day post-fracture. Trizol reagent (1 mL) (Invitrogen, Carlsbad, CA, USA) was added directly to the tissues after being crushed by pestle grinder in liquid nitrogen. The supernatant was collected after centrifugation at 8000 rpm for 5 min. According to commercialized protocol, the phase separation was performed. Total RNA was incubated in 0.5 mL isopropanol at −80 °C overnight. Thereafter it was centrifuged at 12,000 rpm for 10 min at 4 °C. RNA pellet was then washed by 75% ethanol twice and was centrifuged at 7500 rpm for 5 min at 4 °C. Total RNA pellet was put in a sterile hood to be briefly air-dried. Finally, it was dissolved in 100 µL RNase free water to be stored at −80 °C.

### 4.3. MicroRNAs (miRNAs) Microarray Labeling and Hybridization

Each total RNA sample concentration was determined using NanoDrop ND-1000 spectrophotometer (NanoDrop, Wilmington, DE, USA). All RNA samples have 260/280 and 260/230 higher than 1.8 and 1.0 respectively. Samples were labeled and hybridized on Agilent 8 × 60 K Mouse miRNA Microarray (Agilent Technologies, Santa Clara, CA, USA) Release 17.0 according to manufacturer protocol. In brief, exactly 100 ng of RNA sample was used. The 3′ end of RNA was dephosphorylated by calf intestinal phosphatase (Agilent Technologies) and then ligated with pCp-Cy3 (Agilent Technologies) using T4 RNA ligase (Agilent Technologies). Then the labeled samples where hybridized for 20 h at 20 rotations per minute. After hybridization, the arrays were washed and scanned by Agilent scanner and then the image was analyzed by Agilent Feature Extraction 10.7 (Agilent Technologies).

### 4.4. Differentially Expressed miRNAs

The miRNA microarrays’ data were qualified and normalized using limma package on R platform [53]. Two samples were dropped for low data quality (one is adult mice at 0-week, the other is aged mice at 0-week). A total of 16 samples (2 adult mice at 0-week, 3 adult mice at 2-week, 3 adult mice at 4-week, 2 aged mice at 0-week, 3 aged mice at 2-week, 3 aged mice at 4-week) were included in further analysis. Then, miRNAs that significantly differentially expressed in calluses of adult female mice at 2-week and 4-week post-fracture are identified using limma package on R platform [53] with the criterion *p*-value < 0.05 & |log_2_FC (fold change)| ≥ 1 (2-week vs. 0-week, 4-week vs. 0-week, *t*-test). The differentially expressed miRNAs in calluses of aged female mice after fracture were identified in the same method.

### 4.5. Gene Microarray Data

Gene microarray data of mice angiogenesis, chondrogenesis, and osteogenesis was downloaded from the Gene Expression Omnibus (GEO) database (Data id: GDS1631, GDS1632, GDS1633, GSE64141 and GSE7507). Differentially expressed genes in angiogenesis, chondrogenesis, and osteogenesis were identified using limma package on R platform [53] with the criterion *p*-value < 0.05 (*t*-test).

### 4.6. Construction of Protein-Protein Interaction (PPI) Networks

Interactions among products of the differentially expressed genes were identified using protein-protein interaction data downloaded from the BioGRID database [54] (version 3.4.130). Interactions supported by evidence from at least one wet-experiment were selected to construct the PPI networks involved in bone fracture healing. A total of 36,951 PPI interactions were used in further analysis.

### 4.7. Prediction of miRNA-Target Interactions

Experimentally validated miRNA-target interactions were collected from miRTarBase database [55] (Release 6.0). More miRNA-target interactions were predicted using DIANA [56], miRanda [57], miRDB [58], and TargetScan [59]. The miRNA-target interactions supported by evidence from at least one wet-experiment or two prediction methods were selected for further analysis. Since miRNAs target genes and inhibit their expression, the expression pattern of a miRNA and its target gene should be opposite. Therefore, for up-regulated miRNAs in bone fracture healing, down-regulated target genes were selected, while for down-regulated miRNAs, up-regulated target genes were selected. Finally, 43,839 miRNA-target gene interactions were predicted to be involved in bone fracture healing.

### 4.8. miRNA Contribution to Fracture Healing Impairment

The power of the miRNA on the PPI networks (PRN) is calculated as follows:

$$\text{PRN}=\sum_{i=1}^{n_{T}}{\text{PN}}_{i}$$

where $n_{T}$ is the number of all targets of the miRNA and ${\text{PN}}_{i}$ is the number of proteins directly connected to the target protein i in the PPI network of mice.

The miRNA contribution to impaired fracture healing (RCIFH) is calculated as follow:

$$\text{RCIFH}=\left({\text{log}}_{2}^{\frac{E_{\text{NH}i}}{E_{\text{NN}i}}}-{\text{log}}_{2}^{\frac{E_{\text{AH}i}}{E_{\text{AN}i}}}\right){\text{PRN}}_{i}$$

$E_{\text{NH}i}$ is the average value of miRNA *i* expression in adult female mice at 2-/4-week after fracture.
$E_{\text{NN}i}$ is the average value of miRNA *i* expression in adult female mice at 0-week after fracture.
$E_{\text{AH}i}$ is the average value of miRNA *i* expression in aged female mice at 2-/4-week after fracture.
$E_{\text{AN}i}$ is the average value of miRNA *i* expression in aged female mice at 0-week after fracture.
${\text{PRN}}_{i}$ is the power of miRNA *i* in the molecular network of mice.

### 4.9. Pathway Enrichment Analysis

Pathway enrichment analyses were performed with Ingenuity Pathway Analysis (IPA) tools [60] using Fisher’s exact test. Significant enriched pathways for the given genes were identified with the criterion *p*-value < 0.05.

### 4.10. Cell Culture and Transfection

The C3H10T1/2 cell line was obtained from the ATCC (Manassas, WV, USA). These cells were preserved in the complete Dulbecco’s Modified Eagle Medium (DMEM), which was supplemented with 100 U/mL penicillin, 100 mg/mL streptomycin, and 10% fetal bovine serum. These cells were maintained in a humidified 5% CO_2_ atmosphere at 37 °C. Before transfection, C3H10T1/2 cells were placed in 6-well plates at about 10^5^ cell/well. The miR-494 mimics, anti-miR-494, or their inactive controls were transfected into C3H10T1/2 cells according to the manufacturer’s protocol, respectively. After an incubation for 24 h, these cells were trypsinized for further chondrogenic differentiation assay.

### 4.11. Chondrogenic Differentiation Assay

After transfection and 24 h incubation, C3H10T1/2 cells were treated with high density micromass cultures. These cells were then trypsinized by 0.25% trypsin. Then the cells were modulated at the density of 10^7^ cells/mL. Then the suspension was placed into a 12-well plate for 10 µL. Then it was incubated at 37 °C and 5% CO_2_ for 2 h. After that, it was flooded by the chondrogenic differentiation medium in the volume of 1 mL. The chondrogenic differentiation medium was replaced every 2 days. The medium was composed of ascorbate, dexamethasone, sodium pyruvate, proline, Insulin-Transferrin-Sodium selenite (ITS+) Supplement, and Transforming growth factor-β3 (TGF-β3).

### 4.12. Quantitative RT-PCR Analysis

RNeasy Mini Kit (Cat no. 74106, QIAGEN, Hilden, Germany) was used to extract total RNAs from the cultured cells using the commercialized protocol. The cells were collected in a reaction tube and were treated with 700 µL QIAzol. Then they were mixed with 140 µL chloroform. After a 15 min centrifugation at 12,000 rpm at 4 °C, the upper aqueous phase was then transferred to the RNeasy Mini spin column using a 2 mL collection tube, and then was mixed with 100% ethanol. Thereafter, it was washed with 500 µL Buffer RPE and 700 µL Buffer RWT. After that, total RNAs were reverse-transcribed to cDNA using the previously established protocol. The solution contained 1 µL of cDNA product, 5 µL of 2× SYBR^®^ Green Mix, 0.5 µL of each primer and 3 µL nuclease-free water. The fluorescence signal was collected by ABI PRISM^®^ 7900HT System (Applied Biosystems, Foster City, CA, USA).

## 5. Conclusions

Impaired fracture healing in aged females is still a challenge in clinics. In this study, we identified differentially expressed miRNAs during impaired fracture healing in aged female mice. Additionally, we designed a novel RCIFH method to analyze the importance of differentially expressed miRNAs to impaired fracture healing. High RCIFH miRNAs were found to potentially influence the process of angiogenesis, chondrogenesis, osteogenesis, and, especially, osteoclastogenesis in fracture healing. The results might improve our knowledge of impaired fracture healing. Moreover, the RCIFH method would promote the study of miRNAs in impaired fracture healing.

## Figures and Tables

**Table 1:** The top 10 high miRNA contribution to impaired fracture healing (RCIFH) microRNAs (miRNAs) at 2- or 4-week (|log_2_FC| ≥ 1). A positive RCIFH miRNA promotes bone fracture healing, while a negative RCIFH miRNA inhibits it.

2-Week		4-Week	
miRNA	RCIFH	miRNA	RCIFH
miR-494	−1692.98	miR-494	−2038.91
miR-139-5p	−428.294	miR-125a-3p	−492.18
miR-142-5p	−278.332	miR-24	326.6378
miR-206	269.8789	miR-144	−271.429
miR-181b	222.3468	miR-497	259.1864
miR-199a-5p	203.6746	miR-195	246.0054
miR-223	−192.034	miR-15b	230.7585
miR-144	−182.219	miR-23b	168.3962
miR-125a-5p	180.9605	let-7e	143.6061
miR-22	−151.984	miR-223	140.6847

## Supplementary Materials

Supplementary materials can be found at http://www.mdpi.com/1422-0067/17/8/1260/s1.
